# Evaluation of Circulating miRNA Biomarkers of Testicular Germ Cell Tumors during Therapy and Follow-up―A Copenhagen Experience

**DOI:** 10.3390/cancers12030759

**Published:** 2020-03-23

**Authors:** Nina Mørup, Ewa Rajpert-De Meyts, Anders Juul, Gedske Daugaard, Kristian Almstrup

**Affiliations:** 1 Department of Growth and Reproduction, Rigshospitalet, University of Copenhagen, Copenhagen 2100, Denmark; 2 International Center for Research and Research Training in Endocrine Disruption of Male Reproduction and Child Health (EDMaRC), Rigshospitalet, University of Copenhagen, Copenhagen 2100, Denmark; 3 Department of Oncology, Rigshospitalet, University of Copenhagen, Copenhagen 2100, Denmark

**Keywords:** miRNA, miR-371a, AFP, HCG, testicular germ cell cancer, chemotherapy, follow-up, TGCT

## Abstract

New microRNA-based serum biomarkers (miRNA-367-3p, -371a-3p, -372-3p, and -373-3p) have shown great potential for the detection of testicular germ cell tumors (TGCTs), but few studies have investigated the clinical utility and performance of these tests in treatment monitoring. In this study, circulating miRNA levels were measured, together with serum tumor markers alpha-fetoprotein (AFP), β-subunit of human chorionic gonadotropin (β-HCG) and lactate dehydrogenase (LDH) in 406 consecutive blood samples obtained during the treatment and follow-up of 52 TGCT patients at the Copenhagen University Hospital. After testing three different methods of RNA isolation from peripheral blood and PCR quantification in a subset of samples (n = 15), the best performing setup of targeted isolation of miRNAs inside and outside exosomes was selected to analyze all samples. At primary diagnosis, the miRNAs significantly outperformed the serum tumor markers, with a sensitivity and specificity of 78% and 100% (based on 40 patients), respectively. The picture was not as clear when patient trajectories were investigated, with both positive and negative signals for miRNAs and serum tumor markers. To establish whether measuring miRNAs adds value beyond the primary diagnosis, large prospective clinical trials comparing miRNAs and classical tumor markers during the treatment and follow-up of TGCT patients are needed.

## 1. Introduction

Testicular cancer is a rare disease, but nevertheless the most common type of malignancy encountered in young men, with a peak incidence between the age of 15 and 44 [1]. Testicular germ cell tumors (TGCTs) are the most frequent type of testicular cancer, accounting for approximately 95% of all testicular cancers [2]. 

Well established serum tumor markers (STMs) for guiding the management of patients with germ cell tumors include human chorionic gonadotropin (HCG), α-fetoprotein (AFP), and lactate dehydrogenase (LDH) [3]. The magnitude of post-orchiectomy STM elevations is used to stratify risk and select treatment. Rising markers are often the earliest evidence of relapse, and the false-positive rate is low. Serial STM measurements may be needed to determine whether STM levels are rising or falling and, if falling, whether the decline approximates the marker’s biologic half-life (24 to 36 hours for HCG; 7 days for AFP). A major problem is, however, that the STMs are only elevated in 48–60% of TGCT cases at primary diagnosis, irrespective of histological type [4,5,6,7]. 

As an alternative, or in addition to, the STMs, a panel of circulating microRNAs (miR-367-3p, miR-371a-3p, miR-372-3p, and miR-373-3p) has been investigated intensively, due to their high expression in most TGCTs (except teratomas). These miRNAs have been proposed as novel biomarkers that could replace the STMs in the clinical management of patients with TGCTs (for reviews, see Almstrup et al. [8], Lobo et al. [9] and Murray et al. [6]). A recent large multicenter study showed that serum miR-371a-3p had a sensitivity of 90% and specificity of 94% in the initial diagnosis of TGCTs, and hence greatly outperformed STMs [10]. The level of miR-371a-3p correlated with both primary tumor size and clinical stage [10]. However, studies on the clinical utility of the miRNAs during follow-up are sparse. Dieckmann et al. investigated 46 TGCT patients with relapses and found that 38 of such patients had detectable levels of miR-371a-3p, corresponding to a sensitivity of 83%. In their study, treatment caused a significant decrease in the miRNA-371a-3p level in 28 of 29 patients, with pre- and post-treatment samples available [10]. Another study by Mego et al. investigated the level of miR-371a-3p in 180 patients before starting chemotherapy (adjuvant or new line) and detected it in approximately 60% of the patients receiving new line chemotherapy [11]. They found pre-treatment miR-371a-3p levels to be associated with the spread of disease and favorable response to therapy. Rosas Plaza et al. looked at miRNA levels in patients who relapsed after chemotherapy and only 57% had measurable levels of miR-371a-3p [12]. None of these studies followed the patients from primary diagnosis and throughout their treatment course, and hence did not examine the application of these markers in different relevant clinical settings. Only a limited number of complete patient histories have been published so far, e.g., in a study by van Agthoven et al. that presented six cases with a detailed chronology of miRNAs and STMs during their follow-up, relapse and treatment periods, and found the miRNAs to be more sensitive in detecting residual disease and relapse (excluding teratomas), compared with β-subunit of HCG (β-HCG), AFP and LDH [13].

The technical standardization of miRNA-based tests is still a work in progress in several different laboratories, hence, international guidelines of how miRNA measurements should be performed are still lacking. Studies comparing different ways of RNA isolation and the quantification of miRNA levels are needed to provide the objective evidence for best technical set-up, but these are largely missing. 

Here, we investigated the performance of three technically different methods of quantifying the embryonic miRNAs miR-367-3p, miR-371a-3p, miR-372-3p and miR-373-3p. With the best performing setup, we further investigated the utility of these miRNAs during the treatment and follow-up of 52 patients with TGCT.

## 2. Results

### 2.1. Methodological Comparison

**Figure 1:**
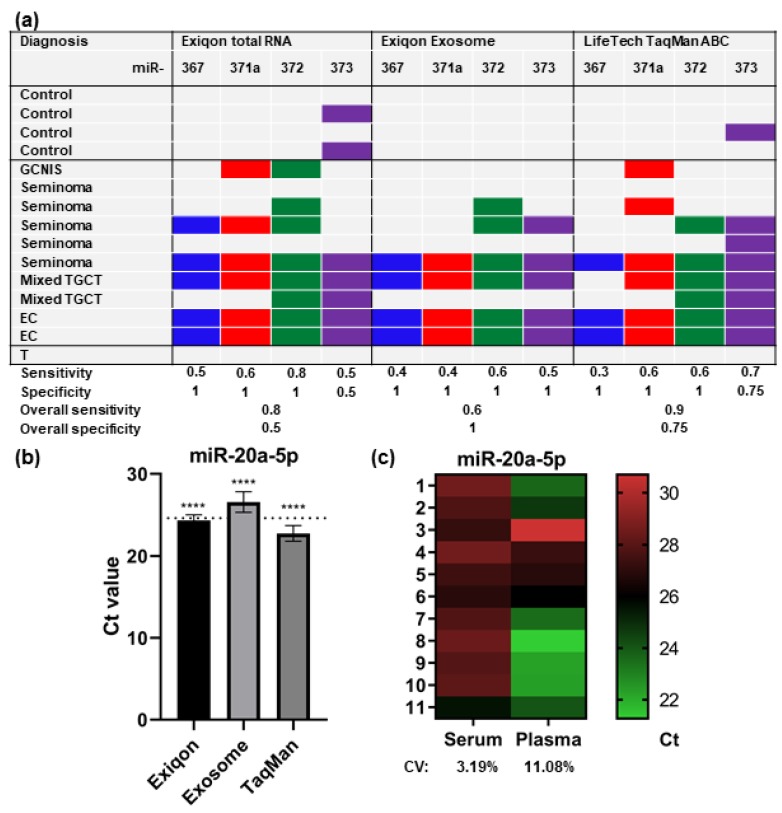
Methodological comparison. (**a**) Comparison of three different methods for detection of circulating miRNAs: Exiqon total RNA purification (miRCURY RNA Isolation Kit), cDNA synthesis (Universal cDNA Synthesis Kit II) and qPCR (PCR ExiLENT SYBR® Green master mix) (Exiqon total RNA); Exiqon exosome purification (miRCURY™ Exosome Isolation Kit—Serum and plasma) followed by total RNA purification (miRCURY RNA Isolation Kit), cDNA synthesis (Universal cDNA Synthesis Kit II) and qPCR (PCR ExiLENT SYBR® Green master mix) (Exiqon Exosome); and TaqMan® miRNA ABC purification kit—Human Panel A, cDNA synthesis (Advanced miRNA cDNA synthesis kit) and qPCR (TaqMan Fast Advanced qPCR Mastermix with corresponding advanced miRNA TaqMan probes) (LifeTech TaqMan ABC). Fifteen samples were analyzed, representing four controls, one GCNIS, five seminoma, two mixed TGCTs, two embryonal carcinomas, and one teratoma. A colored cell indicates that the specific miRNA was detected in that sample by use of the specific method. For sensitivity and specificity calculations, the sample from the teratoma patient was excluded, since teratomas are not expressing the miRNAs. Sensitivity and specificity are shown for each miRNA in each setup, as well as a combined sensitivity and specificity, assuming that the detection of one or more miRNA gives a positive signal. (**b**) Bar plot of the mean Ct value for miR-20a-5p in all samples using the three different methods. The mean Ct values are significantly different from each other (p <0.0001), using a one-way Anova. The mean Ct of all three methods is shown with a dotted line, and the error bars reflect the standard deviation. (**c**) Heatmap showing the miR-20a-5p Ct values in serum and plasma samples using the LifeTech method. The CV in serum was 3.19% and in plasma 11.08%. Abbreviations used: GCNIS; germ cell neoplasia in situ, TGCT; testicular germ cell tumor, EC; embryonal carcinoma, T; teratoma, CV; coefficient of variation.

We compared three different methods of purification and quantification of miRNAs, to select the best assay for miRNA quantification. In the first two methods, RNA was purified by a column-based approach (miRCURY RNA Isolation Kit from Exiqon, Vedbaek, Denmark), either directly by using total plasma (referred to further in the text as Exiqon), or by using exosomes purified from plasma (referred to as Exosome). Both purification methods were followed by cDNA synthesis and qPCR using the Universal cDNA Synthesis Kit II and PCR ExiLENT SYBR® Green master mix from Exiqon. In the third method, miRNAs were purified using magnetic beads with target-specific anti-miRNAs attached (TaqMan® miRNA ABC purification kit—Human Panel A from Life Technologies (Thermo Fischer, Naerum, Denmark), followed by cDNA synthesis by use of the TaqMan^TM^ Advanced miRNA cDNA synthesis kit and qPCR, using TaqMan^TM^ Fast Advanced Master Mix and TaqMan^TM^ Advanced miRNA Assays (referred to as LifeTech, Thermo Fischer Naerum, Denmark). The levels of miR-367-3p, -371a-3p, -372-3p and -373-3p were quantified by the three different approaches in ten patients with TGCTs, one patient with GCNIS and four controls (Figure 1a and Supplementary Table S1). In accordance with previous studies [10,14], one patient with a pure teratoma did not have measurable levels of any of the miRNAs. Overall, the LifeTech method showed the best results, with a sensitivity of 90% and a specificity of 75%. The Exiqon method with (Exosome) and without (Exiqon) prior exosome purification had sensitivities of 80% and 60%, respectively, and specificities of 50% and 100%, respectively. One case with a seminoma had unmeasurable levels of all the miRNAs by use of all three approaches, which was probably due to a very small tumor size in this patient (histologically evaluated to be less than 0.5 mm^2^). When evaluating the levels of the endogenous control miR-20a-5p (miR-20a), the Ct values were significantly lower in the samples purified by use of the LifeTech method, compared with both the Exiqon and Exosome methods (Figure 1b) (*p*-value: <0.0001), even though the input volume was much smaller than the other methods (50 µL plasma with the LifeTech method, 200 µL plasma with the Exiqon method and 600 µL plasma with the Exosome method). The LifeTech method hence yielded a better sensitivity and specificity and higher levels of the endogenous control and was used in all subsequent analyses. With the LifeTech method, we further compared matched serum and plasma samples to test the differences of miR-20a levels between the two. We found that miR-20a was more stable in serum compared with plasma (Figure 1c), with a coefficient of variation (CV) of 3.19% in serum, compared with 11.08% in plasma. In all subsequent analyses, we used serum.

### 2.2. Clinical Performance

**Figure 2:**
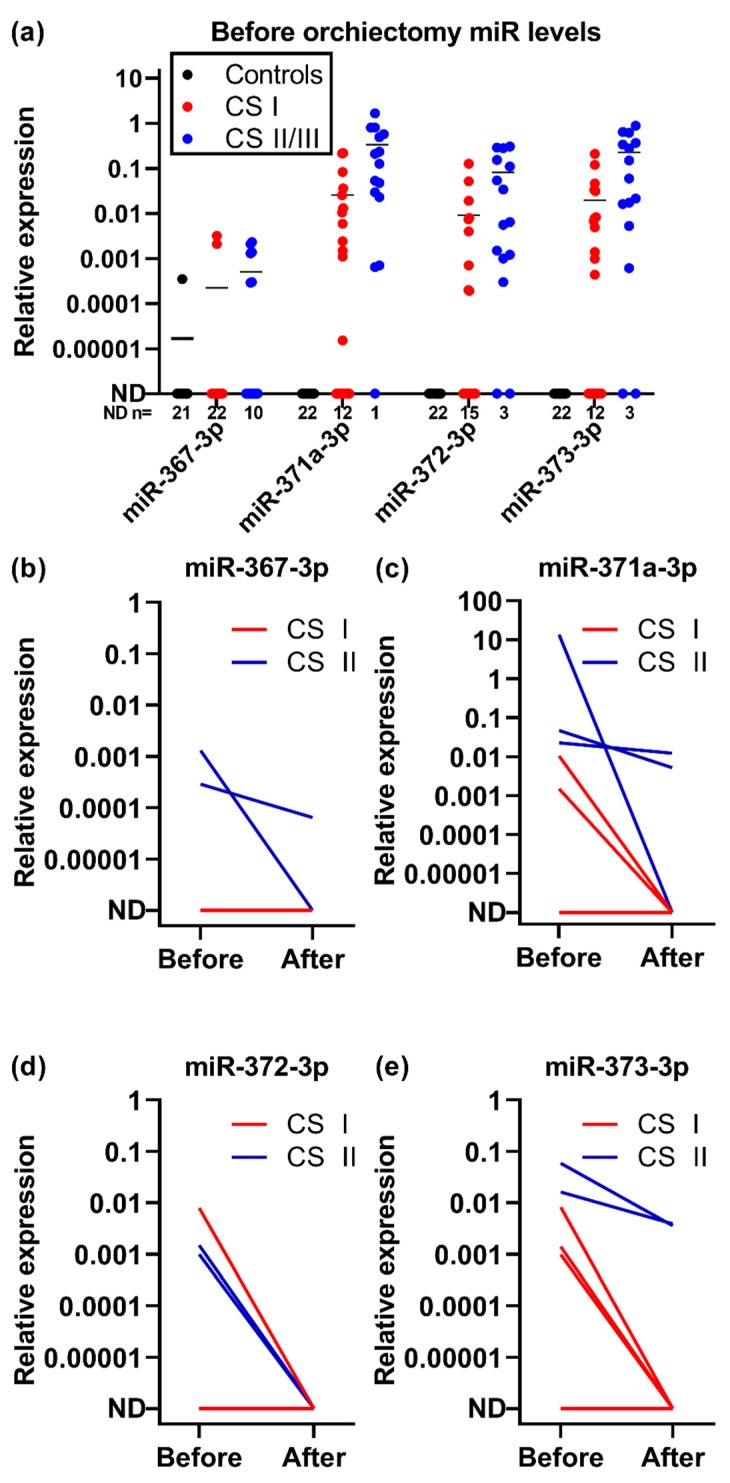
Clinical validation of the LifeTech method. (**a**) Relative miRNA expression normalized to miR-20a-5p in serum from 40 patients, before orchiectomy compared with 22 controls without TGCTs. (**b–e**) matched serum from 8 patients before and after orchiectomy. Blood samples were drawn 1–12 days before orchiectomy (median 3 days) and again 2–21 days after orchiectomy (median 18 days). Red lines represent patients with CSI and blue lines represent patients with CSII. Abbreviations used: TGCT; testicular germ cell tumor, CSI; clinical stage I, and CSII; clinical stage II.

The LifeTech setup was subsequently tested in a larger group of 40 serum samples collected from patients with TGCTs before orchiectomy and 22 controls without TGCTs (Table 1 and Table 2 and Figure 2a). Only one control had elevated levels of miR-367-3p, but none of the controls had measurable levels of any of the other miRNAs (Figure 2a). miR-367-3p was only elevated in eight TGCT patients (20%) and consequently holds very limited clinical value in our setup, hence, we excluded miR-367-3p from the calculations of diagnostic performance. At least one of the other three miRNAs (miR-371a-3p, -372-3p, and -373-3p) was measurable in 31 of 40 patients (19 of 23 non-seminoma (NS) patients and 12 of 17 seminoma (S) patients) (Table 3), giving a sensitivity of 78% and a specificity of 100%. Of the patients with pre-orchiectomy blood samples, 25 patients (62.5%) had clinical stage (CS) I TGCTs (12 NS and 13 S), 14 patients (35.0%) had CS II (10 NS and 4 S) and one patient (2.5%) had CS III (NS) (Table 2 and Table 4). When dividing the patients into groups according to CS, 17 out of 25 CS I patients and 14 out of 15 CS II/III patients had measurable levels of at least one of the three miRNAs (miR-371a-3p, -372-3p, and -373-3p), giving sensitivities of 68.0% and 93.3%, respectively. The miRNA levels were slightly higher in CS II/III compared with CS I patients (Figure 2a). In comparison, β-HCG and AFP (available for 39 patients) combined had a sensitivity of 49% (19 out of 39 patients) for the whole cohort (5 of 16 S patients and 14 of 23 NS patients), 50% (12 of 24 patients) for CS I (3 of 12 S patients and 9 of 12 NS patients) and 46.7% (7 out of 15 patients) for CS II/III (2 of 4 S patients and 5 of 11 NS patients). When combining AFP, β-HCG and the miRNAs, the sensitivities were 85% (33 out of 39 patients) for the whole cohort (12 of 16 S patients and 21 of 23 NS patients), 79.2% (19 of 24 patients) for CS I (9 of 12 S patients and 10 of 12 NS patients) and 93.3% (14 out of 15 patients) for CS II/III (3 of 4 S patients and 11 of 11 NS patients).

Eight patients had blood samples drawn both before and after orchiectomy, five patients with CS I seminoma and three patients with CS II non-seminoma. All cases with measurable levels of miRNAs before orchiectomy experienced a decrease in miRNA levels after orchiectomy (Figure 2b–e), which is in accordance with previous studies [10,15,16]. Only one patient did not have detectable levels of any of the miRNAs before orchiectomy; this was a patient with a CS I seminoma. The five patients with CS I seminomas all had unmeasurable levels of the miRNAs after orchiectomy. The levels of the miRNAs remained detectable after orchiectomy in two patients with CS II non-seminoma, although at a lower level (Figure 2b–e). All three patients with CS II non-seminomas were started on chemotherapy after orchiectomy due to metastatic disease (Figure 2b–e). The patients with the highest levels of miRNA-367-3p, -371a-3p, and -373-3p before orchiectomy had CS II TGCTs (Figure 2b,c,e). One patient with a CS I TGCT had higher pre-orchiectomy levels of miR-372-3p compared with the CS II patients (Figure 2d).

### 2.3. Patient Trajectories

**Figure 3:**
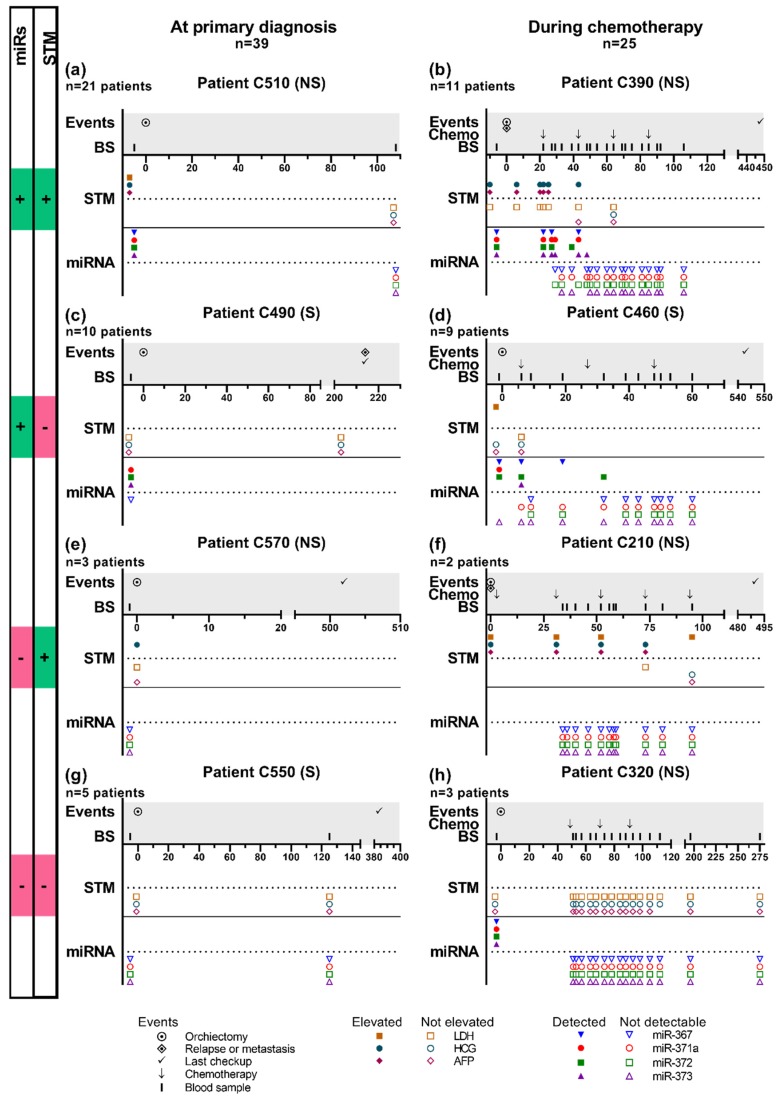
Examples of evolution of miRNAs and serum tumor markers (STMs) at primary diagnosis and during treatment in eight representative patients with TGCT. The number of patients belonging to each group (n) is given under the group symbol (a-h). (**a**,**b**) Patients with measurable levels of both STMs (AFP, β-HCG, and LDH) and miRNAs. (**c**,**d**) Only measurable levels of miRNAs, non-elevated STM levels. (**e**,**f**) Only elevated levels of STM, unmeasurable miRNA levels. (**g**,**h**) Non-elevated levels of STMs and miRNAs. (a,c,e,g) Thirty-nine patients had information on both miRNAs and STM at primary diagnosis/before orchiectomy. (b,d,f,h) Twenty-five patients had information on both miRNAs and STMs during chemotherapy treatment. Abbreviations used: STM; serum tumor markers, NS; non-seminoma, S; seminoma, BS; blood sample, Chemo; chemotherapy, LDH; lactate dehydrogenase, HCG; beta-subunit of human chorionic gonadotropin, AFP; alpha-fetoprotein, miRs; miRNAs.

We measured the level of miRNAs in 406 serum samples from 52 patients with TGCTs. Serum was obtained during their treatment and follow-up period (range: 1–20 samples per patient, median: 5 samples per patient). The tumor components, number of samples, timing of blood sampling and the disease trajectories varied greatly between patients and it is therefore difficult to generalize the results. In Figure 3, we highlight eight examples of patient histories, grouped according to whether the miRNAs and/or STMs were informative at the given time point of their disease. The remaining patient histories can be seen in Supplementary Figure S1. The patients were divided into four groups; group 1 had elevated levels of both miRNAs and STMs, group two had elevated levels of miRNAs but normal STMs, group 3 had unmeasurable levels of miRNAs but elevated STMs and group 4 had unmeasurable levels of miRNAs and normal STMs. 

At primary diagnosis (before orchiectomy), we analyzed samples from 39 patients, with both miRNA and STM levels available (Figure 3a,c,e,g). Of these, 21 patients (54%) belonged to group 1 (Figure 3a), 10 patients (26%) belonged to group 2 (Figure 3c), three patients (7%) to group 3 (Figure 3e) and five patients (13%) to group 4 (Figure 3g).

Thirty-two patients were started on chemotherapy treatment, but three did not have blood samples available for miRNA analysis and four had no information about STMs, leaving 25 patients undergoing chemotherapy with measurements of both miRNAs and STMs (Figure 3b,d,f,h). All patients had standard chemotherapy and obtained complete remission, and there was no evidence of disease/relapse in the follow-up period. Of these, 11 patients (44%) had elevated and measurable levels of STMs and miRNAs, respectively, at some point after starting chemotherapy (group 1, Figure 3b), nine patients (36%) had measurable miRNAs but normal STMs during chemotherapy (group 2, Figure 3d), two patients (8%) had elevated levels of STMs, but unmeasurable miRNAs during chemotherapy (group 3, Figure 3f), and three patients (12%) had normal and unmeasurable levels of STMs and miRNAs throughout chemotherapy, respectively (group 4, Figure 3h).

## 3. Discussion

Diagnosis, treatment and follow-up of patients with TGCT to a large extent depend on the detection and temporal evolution of classical tumors markers, like β-HCG, AFP and LDH. The STMs have been used for decades with good results, however, for a large proportion of TGCT patients, no suitable STMs exist. Several studies have shown that the miRNAs, miR-367-3p, miR-371a-3p, miR-372-3p and miR-373-3p can outperform STMs in diagnostic performance (for review, see Almstrup et al. [8]). In particular, miR-371a-3p has shown great sensitivity in several independent studies [10,13,17]. However, the evidence concerning the performance of miR tests for disease monitoring is still scarce, and only few studies have reported repeated miRNA measurements in the same patients during treatment and follow-up. To our knowledge, our study is the largest of this kind, and the most complete regarding parallel monitoring of miRNA and STM levels throughout patients’ treatment from primary diagnosis, during chemotherapy (if applicable) and through follow-up. We generally found very good performance of miRNAs in the primary diagnosis, which is in line with previous studies. Quite unexpectedly, however, we found that results obtained during treatment and follow-up were not clear-cut. 

Some patients have normal/unmeasurable levels of both STMs and miRNAs. As noted during our methodological optimization, this could represent small tumors that presumably do not release high enough levels of circulating miRNA (and/or STMs) to be detected. In general, the miRNAs were more sensitive than the STMs and several more patients could potentially have been discovered at primary diagnosis (26%) if the miRNA measurements were included in the clinical routine.

The sensitivity and specificity of our assay were not as high as reported in other larger studies, e.g. Dieckmann et al. 2019 (sensitivity of 78% vs. 90% for miR-371a-3p) [10]. This could be due to the smaller study population in our study, but differences in the number of patients with different clinical stages could also bias the comparison between studies. Our study included many CS I patients (30 (57.7%) vs. 21 (40.4%) CS II/III), who have previously been reported to have lower levels of miR-371a-3p compared with CS II/III [10]. In accordance, we observed a lower sensitivity for the miRNAs for CS I patients (68%) than for CS II patients (92.9%). The sensitivity for CS I patients is, however, much lower in our study than in the study by Dieckmann [10], who reported a sensitivity of 83.4% for this patient group, so the clinical stage alone is not an explanation for the lower sensitivity found in our study. Combining the STMs and miRNAs increased the sensitivity to 85% (75% for CS I and 93.3% for CS II/III), indicating that both STMs and miRNAs can contribute to the diagnosis of TGCTs. Albeit, the slightly lower sensitivity of miR-371a-3p in our assay, miR-371a-3p was, in accordance with previous studies, found to have the highest diagnostic performance of all the tested miRs. The discovery rate of TGCT patients positive for any of the miRs was also lower than previously reported [10,13], and especially miR-367-3p, showed a lower performance in our study compared to earlier studies [13]. This could be due to differences in RNA purification methods, technical differences in the assay (e.g., of miR-367-3p), besides the abovementioned differences in the distribution of clinical stages. To better compare studies across different populations and the diagnostic performance of different labs, international guidelines and standardization of the assays are needed. Additionally, common spike-in controls and endogenous miRNA controls would facilitate direct comparisons between labs and the applied methodology [8]. Furthermore, we chose a cut-off value of Ct = 40, which is practical in a clinical routine with day-to-day analysis. In other studies, ROC curves and Youden’s index have been used to determine the most optimal cut-off for the entire set of samples included in the study [10]. Such thresholds, however, depend on the set of samples analyzed, as well as the technical setup, and is therefore less practical in a clinical routine setting.

In our study, we only had eight patients, with both pre-orchiectomy and post-orchiectomy (within 21 days after orchiectomy) samples available. Previous studies have shown that miRNAs have a very short half-life in circulation and that successful orchiectomy leads to unmeasurable miRNA levels within days [10,18], thus we would expect that the miRNA levels would have decreased to unmeasurable levels in all patients with localized disease and only miRNA levels in patients with disseminated disease would remain elevated. This was also seen when miRNA levels in all patients with localized disease (CS I) dropped to unmeasurable levels after orchiectomy and the two patients with remaining elevated miRNA levels had metastatic disease (CS II) (Figure 2a). 

The most important diagnostic gain of using miRNAs is in patients with seminomas, since β-HCG is only elevated in a subset of these patients and LDH is not a reliable tumor marker (30–50% [7,10]). Accordingly, in our study, the STMs showed a sensitivity of 31% for diagnosing primary seminomas, whereas the miRNAs (miR-371a-3p and/or miR-372-3p and/or miR-373-3p) had a sensitivity of 71%. In combination, the miRNAs and/or STMs had a sensitivity of 75%. These results clearly indicate that the miRNAs can improve the diagnosis of patients with seminomas, but we need data to show efficacy in treatment monitoring and the usefulness in detecting disease progression and relapse.

Based on our results, the added value of measuring miRNAs is largest at primary diagnosis. miRNAs seem to disappear from the circulation within days after starting treatment for disseminated disease and whether miRNAs contribute to evaluation of the patients’ disease during chemotherapy and subsequent follow-up is, at the moment, very unclear. The follow-up time for the clinical cohort was limited, since the patients had their orchiectomy between 2016 and 2018. However, the majority of relapses will be diagnosed within the first year of follow-up [19,20]. Until more evidence is available from larger clinical trials, we suggest that both STMs and miRNAs are measured during treatment and follow-up, in centers that have the possibility of conducting prospective studies. 

Differences in the technical protocols can affect the diagnostic performance of the miRNAs. The assay we found to perform best is very similar to the assays applied in other labs. Differences include the method of miRNA purification and the method of cDNA synthesis. With regard to the miRNA purification, Dieckmann et al. used the miRNeasy Mini Kit from Qiagen [17] and van Agthoven et al. used the same protocol as we did [13]. Regarding the cDNA synthesis, in our assay we reverse-transcribe all miRNAs into cDNA, whereas the assays used by e.g. the Looijenga group and the Dieckmann group only reverse-transcribe specific miRNAs of interest, with primers specific for each miRNA. For the miRNA assays to be used in the clinic, it is important to standardize the assay on an international level and to provide quality control programs across labs. It should be decided whether serum or plasma are used in the assay, and what is the most appropriate endogenous control. We found serum to give stable measurements of the endogenous control miR-20a-5p, although the Ct values were higher compared with plasma. Lobo et al [21] found miR-30b-5p levels, another endogenous control, to be higher in plasma compared with serum, but found miR-371a-3p levels to be higher in serum than in plasma. A potential bias when comparing miRNA levels in serum and plasma is the possible heparin contamination seen when using blood sampling tubes coated with heparin for plasma collection. Glinge et al. previously showed that miRNA levels in plasma collected in lithium-heparin coated tubes were unmeasurable and miRNAs in plasma collected in other tubes or in serum were measurable. However, they used a column-based RNA extraction method (NucleoSpin®) [22]. Thus, heparin may influence the miRNA levels measured in plasma samples, at least when using this method of purification. Lobo et al. recently showed that heparin contamination of plasma samples did not influence the Ct value of measured miRNAs from plasma samples, if bead-capture RNA extraction was used, but it did increase Ct values if a column-based RNA extraction procedure (miRNeasy) was used [21]. This increase in Ct could be reversed if heparinase was used. Therefore, heparin contamination should not influence the miRNA levels in our comparison of serum and plasma samples, where we used the bead-capture approach for miRNA isolation.

Serum seems to be the preferred media in most other studies [10,13,23], although both serum and plasma can be used for miRNA measurements, especially if bead-captured miRNA purification is used or heparinase is used [18]. Furthermore, our data indicate that in plasma samples, the miRNAs are present both within and outside of exosomes. The detection of miRNAs was lost in some cases when exosomes were isolated before RNA purification, but most miRNAs were still measurable. As the input volume using the exosome method is 12x higher compared with the LifeTech method (600 µL vs 50 µL), a substantial added value is obtained by including small RNAs that are outside exosomes and not isolating the exosomes first. This is further substantiated by higher Ct values for the endogenous control, miR-20a-5p, when exosomes were isolated, and indicates an overall lower recovery of miRNAs when only isolating exosomal miRNAs.

## 4. Materials and Methods 

### 4.1. Study Design and Participants

#### 4.1.1. Methodological Comparison

A retrospective study was used, which detailed 15 stored plasma samples from patients attending the Department of Growth and Reproduction, Copenhagen University Hospital, Denmark, for the cryopreservation of semen, before undergoing orchiectomy following a diagnosis of testicular cancer or treatment for other diseases (controls) in 2014. Information about diagnosis and germ cell cancer subtype was noted (Supplementary Table S1).

For the comparison of miRNA levels in serum and plasma, matched serum and plasma was collected from 11 patients attending the Department of Growth and Reproduction in 2017. 

#### 4.1.2. Clinical Study of Tumor Markers during Treatment and Follow-Up

This is a prospective study recruiting patients from Copenhagen University Hospital, Denmark in 2017–2019―either attending the semen bank at the Department of Growth and Reproduction for cryopreservation of semen before starting treatment for their various diseases including testicular cancer, or visiting the Department of Oncology to receive chemotherapeutic treatment for their testicular cancer and/or for regular follow-up visits. A total of 74 men were included in this study, including 52 patients with germ cell tumors and 22 controls (Table 1). Of the patients with germ cell tumors, 14 men experienced relapse and 32 were treated with chemotherapy. The controls were men referred to the semen bank, due to the surveillance for testicular pathology or before starting treatment that could affect testicular function, but without a diagnosis of TGCT.

Table 2 shows the number of patients who had blood samples drawn at the specific timepoints during their cancer treatment at the hospital. Of the patients with TGCTs, 40 men had preoperative serum samples and of these, 8 men had both preoperative (1–12 days before orchiectomy) and postoperative (2–21 days after orchiectomy) serum samples drawn. Some of the TGCT patients receiving chemotherapy had repeated samples drawn during the period (25 out of 32 patients given chemotherapy), ranging from 1 to 20 samples per patient.

The following clinical parameters were registered: date of blood aspiration, tumor histology, clinical stage, serum levels of classic tumor markers, chemotherapy received, orchiectomy and/or other surgical interventions.

### 4.2. Ethics

All participants were included after informed consent and signed an informed consent form. The study was approved by the Danish ethical committee (H-16019637) and the Danish data security agency (RH-2016-349) and was performed according to the Helsinki declaration.

### 4.3. Laboratory Methods

#### 4.3.1. Handling of Blood Samples

Whole-blood samples were collected in non-coated tubes, left to coagulate and then spun at 1900× *g* for 10 min at 4 °C, to separate serum from the coagulate. Serum was transferred to Eppendorf tubes and stored at −80 °C until further use. For plasma samples, whole-blood was collected in heparin-coated blood sample tubes and centrifuged at 1900× *g* for 10 min at 4 °C. Plasma was transferred to Eppendorf tubes and stored at −80 °C.

#### 4.3.2. miRNA Measurements

We tested three different commercial kits to purify and quantify circulating miRNAs. We compared RNA isolation methods based on beads coated with anti-miRNAs (TaqMan^TM^ miRNA ABC purification kit, Life Technologies (Thermo Fischer,Naerum, Denmark), total small RNA isolation (miRCURY RNA Isolation Kit, Exiqon, Vedbaek, Denmark), and exosome isolation (Exiqon miRCURY™ Exosome Isolation Kit, Exiqon, Vedbaek, Denmark). Subsequently, two different RT-qPCR systems were tested, the TaqMan^TM^ Fast Advanced kit and the Exiqon miRCURY LNA Universal RT microRNA kit. In general, the manufacturers protocols were followed as outlined below.

#### 4.3.3. Life Technologies

miRNAs were isolated from 50 µL serum by use of the TaqMan^TM^ miRNA ABC purification kit —Human Panel A, according to the manufacturer’s instructions. Before purification, 2 µL of 1 nM endogenous control cel-miR-39 was added to the samples. The purified miRNAs were reversely transcribed into cDNA by use of the TaqMan^TM^ Advanced miRNA cDNA synthesis kit according to the manufacturer’s instructions. Briefly, the cDNA synthesis protocol consisted of a Poly(A) tailing reaction, a ligation reaction adding an adaptor sequence, a reverse transcription reaction and a pre-amplification reaction. The quantification of the miRNAs was done by use of TaqMan^TM^ Fast Advanced Master Mix and TaqMan^TM^ Advanced miRNA Assays (hsa-miR-20a-5p, cel-miR-39-3p, hsa-miR-367-3p, hsa-miR-371a-3p, hsa-miR-372-3p, and hsa-miR-373-3p), according to the manufacturer’s instructions. All samples were run in duplicates or triplicates. miR-20a-5p was used as an endogenous control for normalization purposes [24] and cel-miR-39-3p was used as a control for purification efficiency. The qPCR was run on Quantstudio 3 (96-well format) or Quantstudio 5 (384-well format) (both gave similar results in direct comparisons). The raw data were processed with Thermo Fischer QuantStudio Cloud. Cq values were used for further analyses.

miRNAs isolated from TCAM2 cells (1 × 10^6^ cells per purification) were used as positive controls and as a measure of cDNA synthesis efficacy, by adding serial dilution series (10× dilutions) for all miRNAs.

#### 4.3.4. Exiqon Exosome Isolation and miRNA Purification

Exosomes were isolated from blood plasma by use of the miRCURY™ Exosome Isolation Kit―Serum and Plasma from Exiqon using 600 µL plasma, according to the manufacturer’s instructions. The isolated exosomes were resuspended in 270 µL resuspension buffer and used for miRNA isolation by use of the miRCURY RNA Isolation Kit, according to the manufacturer’s modified instructions for isolated exosomes, adding 1 µL spike-in and 1.25 µL carrier-RNA to the isolated exosomes before purification. The miRNAs were eluted in 100 µL RNase-free H2O and stored at −80 °C.

#### 4.3.5. Exiqon miRNA Purification

miRNAs were isolated from 200 µL plasma by use of the miRCURY RNA Isolation Kit according to the manufacturer’s instructions, adding a 1 µL spike-in and 1.25 µL carrier-RNA before purification. The miRNAs were eluted in 50 µL RNase free H2O and the purified miRNAs were stored at −80 °C.

#### 4.3.6. Exiqon miRCURY LNA^TM^ Universal RT microRNA PCR

The cDNA synthesis and real-time PCR amplification were done with the Universal cDNA Synthesis Kit II from Exiqon, according to the manufacturer’s instructions from 2014. Then, 2 µL purified miRNAs were mixed with 0.5 µL RNA spike-in before reverse transcription.

A real-time PCR amplification was done, using PCR ExiLENT SYBR® Green master mix (Exiqon) with PCR primer mix (UniSp6, miR-20a, miR-367, miR-371, miR-372, and miR-373), according to the manufacturer’s instructions and it was run on a MX3000. Raw data were processed using the Stratagene software MxPro v4.10 (Agilent, Santa Clara, CA, USA). Average Ct values were used for further analyses.

#### 4.3.7. Measurement of Serum Tumor Markers

The measurement of STMs was done according to standard laboratory guidelines at Copenhagen University Hospital and collaborating hospitals in Denmark.

LDH was measured by enzymatic determination using heparin plasma, by use of the Cobas analyzer, with the Cobas LDHI2 kit (cat. no. 5169330190), with a maximum CV of 5%. β-HCG was measured by Sandwich Electrochemiluminescense Immunoassay (ECLIA), using heparin plasma by use of the Cobas analyzer with the Cobas HCG-β kit (cat. no. 7251025 190), with a maximum CV of 5%. AFP was measured by ECLIA using heparin plasma by use of the Cobas analyser with the Cobas AFP kit (cat. no. 07026706 190), with a maximum CV of 6%. 

### 4.4. Data Analysis:

Relative expression was calculated by the delta-delta-Ct method, using a cut-off value at Ct = 40 and miR-20a-5p as endogenous control [24,25] and normalizer (2-(Target miR - miR-20a)). The sensitivity was calculated by dividing the number of true positives with the total number of predicted positives (true positives + false negatives), assuming that all patients with TGCT, except those with pure teratoma histology, should be positive for the miRNAs and STMs. The specificity was calculated by dividing the number of true negatives with the total number of predicted negatives (true negatives + false positives), assuming that all controls should be negative for the miRNAs and STMs.

GraphPad Prism 8.0.2 was used for the plotting and statistical analyses of the data. A one-way ANOVA with Tukey’s correction for difference between methods was used (miR-20a-5p with three setups). 

## 5. Conclusions

Detection assays that include the targeted isolation of miRNAs found both within and outside exosomes showed the best diagnostic performance in this comparative study of several different laboratory protocols. Measurements of specific miRNAs (miR-371a-3p, -372-3p and -373-3p) provide a significant added value to the primary diagnosis of TGCT, especially for patients with seminomas, and we therefore suggest that the miRNA test should be added to the panel of routine diagnostic markers. Measurements of repeated samples during treatment and follow-up did not reveal a clear advantage of measuring miRNAs over AFP and β-HCG alone. Hence, we still suggest that both appropriate STMs and miRNAs are measured and compared during the treatment and follow-up of patients with a TGCT as part of clinical trials, until the added clinical value of miRNA measurement is clarified.

## Figures and Tables

**Table 1:** Number and characteristics of patients from the clinical cohort, including collected blood samples at different time points.

Clinical Cohort	Number of Cases (%)
Controls	22 (100%)
TGCT patients	52 (100%)
GCNIS	1 (1.9%)
S	23 (44.2%)
NSs	28 (53.8%)
Mixed	24 (46.2%)
EC	3 (5.8%)
C	1 (1.9%)
T	-
Unknown	1 (1.9%)
STAGE	
GCNIS	1 (1.9%)
CS I	30 (57.7%)
CS II	18 (34.6%)
CS III	3 (5.8%)
Prognostic group CS II/III	21
Good	17 (81.0%)
Intermediate	3 (14.3%)
Poor	1 (4.8%)
Patients with relapse	14
Patients receiving chemotherapy	32

TGCT: testicular germ cell tumor, GCNIS: germ cell neoplasia in situ, S: seminoma, NS: non-seminoma, EC: embryonal carcinoma, C: choriocarcinoma, T: teratoma, CS: clinical stage.

**Table 2:** Number of patients with miRNA or STM measurements at specific time points over the course of their disease.

Clinical Cohort	Number of Patients with miRNAs Measured (%)	Number of Patients with STMs Measured (%)
Before orchiectomy	40	39*
CS I	25 (62.5%)	24 (61.5%)
CS II	14 (35.0%)	14 (35.9%)
CS III	1 (2.5%)	1 (2.6%)
Before and after orchiectomy	8	8 (100%)
CS I	5 (62.5%)	5 (62.5%)
CS II	3 (37.5%)	3 (37.5%)
Patients with relapse	3/14 (21.4%)	14
Patients receiving chemotherapy	29/32 (90.6%)	

*: no measurements of STMs were available for the last patient. CS: clinical stage, STM: serum tumor marker.

**Table 3:** Number of patients with measurable or elevated levels of each separate miRNA or STM at primary diagnosis, according to either clinical stage or histological subtype.

Tumor Markers	All Patients	CS I	CS II	CS III	S	NS
Number of patients with miRs measured	40	25	14	1	17	23
At least one measurable miRNA	31 (77.5%)	17 (68%)	13 (92.9%)	1 (100%)	12 (70.6%)	19 (82.6%)
miR-367-3p	8 (20%)	2 (8%)	6 (42.9%)	0	1 (5.9%)	7 (30.4%)
miR-371a-3p	27 (67.5%)	13 (52%)	13 (92.9%)	1 (100%)	9 (52.9%)	18 (78.3%)
miR-372-3p	22 (55%)	10 (40%)	12 (85.7%)	0	7 (41.2%)	15 (65.2%)
miR-373-3p	25 (62.5%)	13 (52%)	12 (85.7%)	0	10 (58.8%)	15 (65.2%)
Number of patients with STMs measured	39	24	14	1	16	23
β-HCG and/or AFP	19 (48.7%)	12 (50%)	7 (50%)	0	5 (31.3%)	14 (60.9%)
LDH	12 (30.8%)	5 (20.8%)	7 (50%)	1 (100%)	6 (37.5%)	6 (26.1%)
β-HCG	18 (46.2%)	11 (45.8%)	7 (50%)	0	5 (31.3%)	13 (56.5%)
AFP	11 (28.2%)	7 (29.2%)	4 (28.6%)	0	2 (12.5%)	9 (39.1%)

Percentage of positive patients is the same as the sensitivity of the miRNA or STM in the given group of patients. Abbreviations: CS: clinical stage, S: seminoma, NS: non-seminoma, miR: miRNA, STM: serum tumor marker, β-HCG: beta-subunit of human chorionic gonadotropin, AFP: alpha-fetoprotein, LDH: lactate dehydrogenase.

**Table 4:** Number of patients with at least one miR detected, AFP and/or β-HCG elevated and miR and/or AFP/HCG at primary diagnosis according to clinical stage and further subdivided into histological type.

Clinical Stage and Histology	miR Detected	AFP and/or β-HCG Elevated	miR Detected and/or AFP Elevated and/or β-HCG Elevated
All patients at primary diagnosis	31/40 (77.5%)	19/39 (48.7%)	33/39 (84.6%)
S	12/17 (70.6%)	5/16 (31.3%)	12/16 (75.0%)
NS	19/23 (82.6%)	14/23 (60.9%)	21/23 (91.3%)
CS I	17/25 (68%)	12/24 (50%)	19/24 (79.2%)
S	9/13 (69.2)	3/12 (25.0%)	9/12 (75%)
NS	8/12 (66.7%)	9/12 (75%)	10/12 (83.3%)
CS II	13/14 (92.9%)	7/14 (50%)	13/14 (92.9%)
S	3/4 (75%)	2/4 (50%)	3/4 (75%)
NS	10/10 (100%)	5/10 (50%)	10/10 (100%)
CS III	1/1 (100%)	0/1 (0%)	1/1 (100%)
S	-	-	-
NS	1/1 (100%)	0/1 (0%)	1/1 (100%)

CS: clinical stage, S: seminoma, NS: non-seminoma, β-HCG: beta-subunit of human chorionic gonadotropin, AFP: alpha-fetoprotein, LDH: lactate dehydrogenase

## Supplementary Materials

The following are available online at https://www.mdpi.com/2072-6694/12/3/759/s1, Figure S1: Complete patient histories of the remaining patients of the study with both miRNA and STM measurements, Table S1: Patient characteristics of methodological optimization cohort.
