# Supplementary material for: Evaluation of Circulating miRNA Biomarkers of Testicular Germ Cell Tumors during Therapy and Follow-up―A Copenhagen Experience
Source: Cancers (Basel). 2020 Mar 23;12(3):759. doi: 10.3390/cancers12030759 (PMC7140092; doi:10.3390/cancers12030759)
Supplement: Supplementary file 1 [file cancers-12-00759-s001.pdf]

cancers

MDPI

Supplementary Materials

Table 1. Patient characteristics of methodological optimization cohort.

Clinical cohort	MEthodological
	Number of cases (%)
Controls	4 (100%)
Healthy	1 (25%)
Inflammation	2 (50%)
Non-GCT TC	1 (25%)
tgct patients	11 (100%)
gcnis	1 (9.1%)
s	5 (45.5%)
ns	5 (45.5%)
Mixed	2 (18.2%)
EC	2 (18.2%)
C	-
T	1 (9.1%)

(T)GCT: (testicular) germ cell tumour, TC: testicular cancer, GCNIS: germ cell neoplasia in situ, S: seminoma, NS: non-seminoma, EC: embryonal carcinoma, C: choriocarcinoma, T: teratoma.

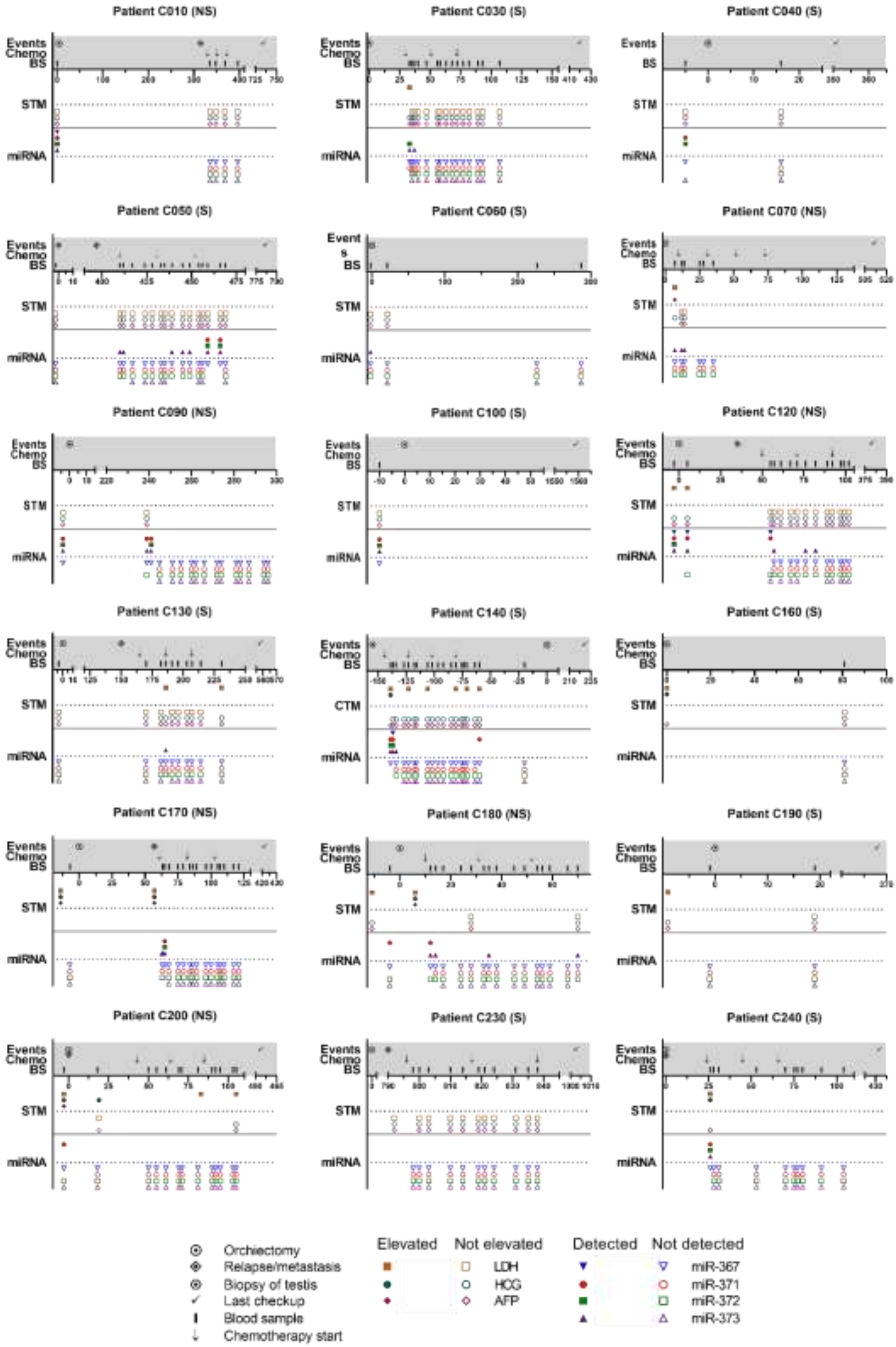
Patient C010 (NS)
Patient C030 (S)
Patient C040 (S)
Patient C050 (S)
Patient C060 (S)
Patient C070 (NS)
Patient C090 (NS)
Patient C100 (S)
Patient C120 (NS)
Patient C130 (S)
Patient C140 (S)
Patient C160 (S)
Patient C170 (NS)
Patient C180 (NS)
Patient C190 (S)
Patient C200 (NS)
Patient C230 (S)
Patient C240 (S)
Events
Chemo
BS
STM
CTM
miRNA
Orchiectomy
Relapse/metastasis
Biopsy of testis
Last checkup
Blood sample
Chemotherapy start
Elevated
Not elevated
LDH
HCG
AFP
Detected
Not detected
miR-367
miR-371
miR-372
miR-373

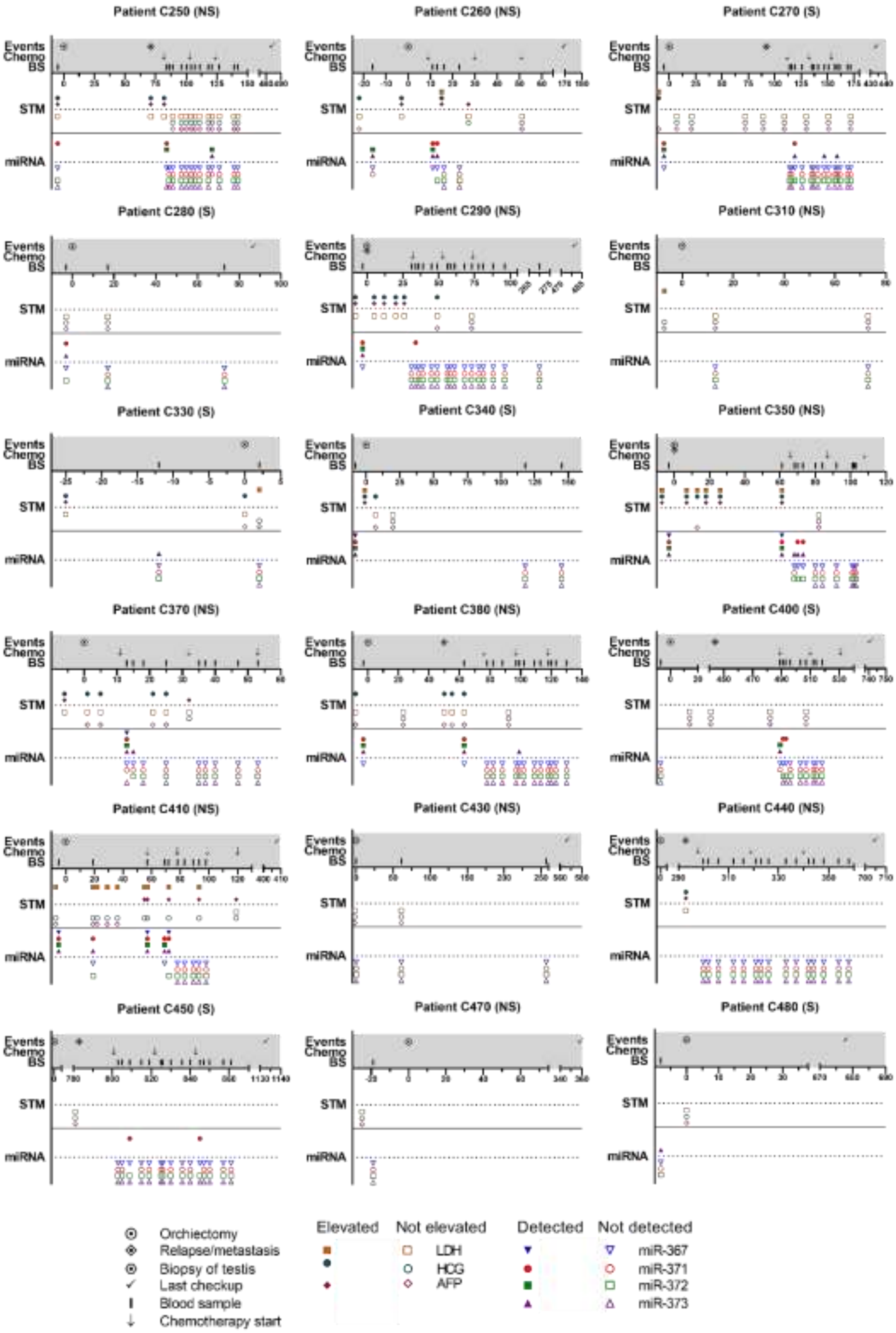
Patient C250 (NS)
Patient C260 (NS)
Patient C270 (S)
Patient C280 (S)
Patient C290 (NS)
Patient C310 (NS)
Patient C330 (S)
Patient C340 (S)
Patient C350 (NS)
Patient C370 (NS)
Patient C380 (NS)
Patient C400 (S)
Patient C410 (NS)
Patient C430 (NS)
Patient C440 (NS)
Patient C450 (S)
Patient C470 (NS)
Patient C480 (S)
Events
Chemo
BS
STM
miRNA
Orchiectomy
Relapse/metastasis
Biopsy of testis
Last checkup
Blood sample
Chemotherapy start
Elevated
Not elevated
LDH
HCG
AFP
Detected
Not detected
miR-367
miR-371
miR-372
miR-373

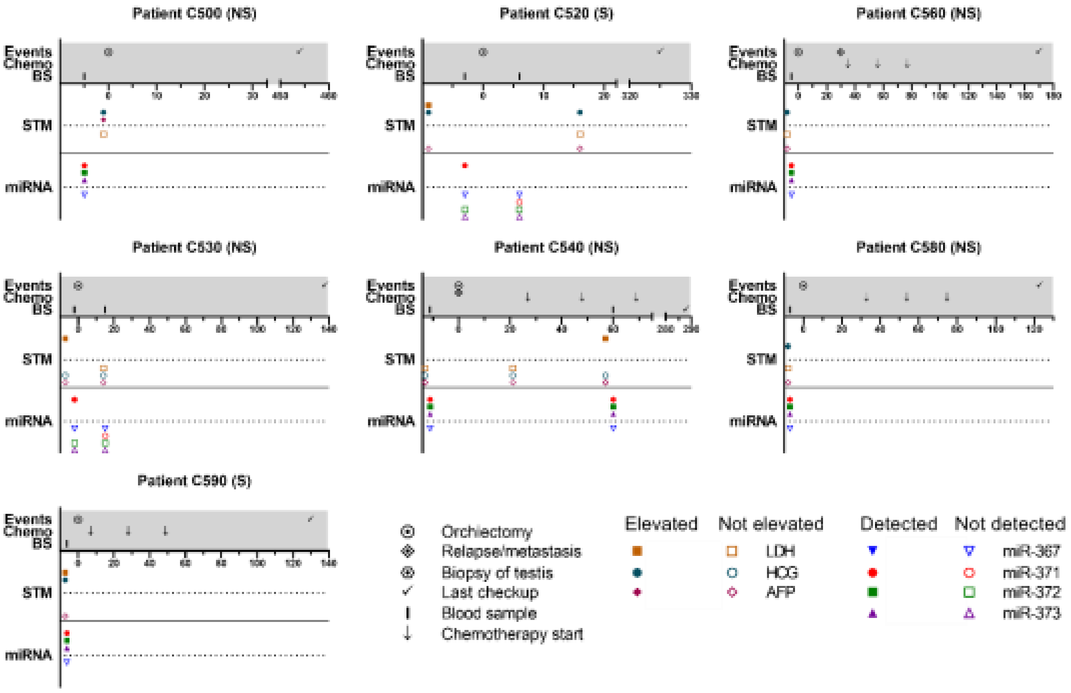

Figure 1. Complete patient histories of the remaining patients of the study with both miRNA and STM measurements. Patient with GCNIS not included in figure, since the patient did not undergo orchiectomy. Abbreviations used: STM; serum tumor markers, NS; non-seminoma, S; seminoma, BS; blood sample, Chemo; chemotherapy, LDH; Lactate dehydrogenase, HCG; beta-human chorionic gonadotropin, AFP; alpha-fetoprotein, miRs; miRNAs.
